# Toward sustainable healthy living in South Africa: the asymmetric effects of clean energy, natural resources and institutional quality

**DOI:** 10.3389/fpubh.2026.1800799

**Published:** 2026-05-28

**Authors:** Paul Terhemba Iorember, Ashley van Niekerk

**Affiliations:** 1 Institute for Social and Health Sciences, University of South Africa, Johannesburg, South Africa; 2 Violence, Injury and Social Asymmetries Research Unit, South African Medical Research Council and University of South Africa, Cape Town, South Africa

**Keywords:** clean energy, health, institutional quality, natural resources, quality of life, South Africa

## Abstract

**Introduction:**

Over the last decade, South Africa has prioritized infrastructure development, focusing on an energy transition and resource efficiency as key areas of reform. However, while the country has reported some socio-economic benefits, it continues to deal with significant health issues, which impose a substantial cost in terms of DALYs and quality of life. This study therefore examines the asymmetric effects of access to clean energy, natural resource endowment, and institutional quality on sustainable, healthy living outcomes (DALYs and quality of life) in South Africa. The study also considers the moderating role of institutional quality.

**Methods:**

The study employed quantile regression and non-parametric Granger causality in quantiles on quarterly data covering the period from Q1 2000 to Q4 2022.

**Results and discussion:**

The findings suggest that access to clean energy is the most significant factor in reducing DALYs from all causes and improving quality of life, with the greatest benefits realized by population groups with relatively low DALYs. Natural resource endowment has no direct effect on DALYs or quality of life, indicating the difficulty of converting resource riches into welfare advantages. Institutional quality has an important, albeit asymmetric, direct effect. Furthermore, the results confirm the moderating role of institutional quality in reducing DALYs among moderately to highly vulnerable population groups. These findings are corroborated by the robustness tests. They suggest that the South African government and its partners should accelerate the transition to clean energy by increasing investment in clean energy resources and/or providing incentives and subsidies. They also show the importance of effective institutions to ensure the health and environmental benefits of energy systems and natural resource management.

## 1 Introduction

Sustainable healthy living is a key feature of the global development agenda, as demonstrated by Sustainable Development Goal 3 (SDG 3), which aims to promote healthy living and wellbeing for people of all ages (61). This goal involves reducing premature deaths, controlling communicable and non-communicable diseases, and improving quality of life, mental health, and environmental health. Disability-adjusted life years (DALYs) and quality of life (QTYL) indicators have become important metrics for tracking sustainable healthy living because they demonstrate both life expectancy and quality of life. Whereas DALYs quantify the burden of disease, QTYL reflects individuals' overall life satisfaction, functional status, psychological wellbeing, and ability to participate meaningfully in social and economic life. DALYs combine years of life lost due to premature death and years lived with a disability into a single figure that illustrates the extent of health loss. Each year, hundreds of millions of DALYs are lost to a mix of communicable, non-communicable, and injury-related causes (1). Quality of life (QTYL) captures the broader dimensions of human wellbeing beyond clinical health outcomes. In public health research, QTYL is often conceptualized through multidimensional frameworks that incorporate physical health, mental health, social relationships, environmental safety, and access to essential services (2). These dimensions are especially relevant in regions like Africa, where inequality, socio-economic challenges, poverty constrained energy access, environmental stressors and governance challenges continue to influence health and wellbeing (3, 4).

Beyond, energy and environmental conditions, spatial accessibility to healthcare services also plays a critical role in shaping health outcomes. Differences in population distribution, healthcare resourcing, and transportation infrastructure determine the ease with which individuals can access essential health services (5), which could influence disease burden and quality of life. This indicates that sustainable healthy living is not only a function of energy systems and institutions, but also of spatial and infrastructural systems that condition access to care. More so, the design of fiscal institutions and public expenditure allocation is critical. Jin et al. (6) show that excessive and insufficient expenditure decentralization can potentially affect welfare and sustainable outcomes.

South Africa is a pertinent and interesting case study. The country has made significant investments in developing its health, socio-institutional and energy systems, which are argued to be central to the promotion of its socio-economic development and ultimately population health (7). Theoretically, the Grossman (8) health production framework posits that population health outcomes are produced through a combination of public inputs such as energy infrastructure which is a major factor of energy access, environmental quality, and institutional capacity, alongside health investments. In this context, access to clean energy directly enhances health by reducing environmental pollutants and improving health outcomes (9). The access to modern and clean energy contributes to residents' wellbeing, especially among those living in rural locations (10).

In the last decade South Africa has prioritized infrastructural development, including its community, housing and energy systems, with energy considered as especially crucial for the country's socio-economic progress, and the key reform that ultimately aims to ensure reliable, affordable, and sustainable electricity supply (55). However, despite these structural investments and reforms, South Africa is still dealing with a significant health burden, including HIV/TB, chronic non-communicable diseases, accidents and other issues, which together impose a significant DALY cost (11), and lower life quality.

In addition, despite its health burden, South Africa has an infrastructure that is increasingly well placed to support its population health, including a rich endowment of natural resources, such as coal, minerals and, increasingly renewable energy potential (solar and wind) (12, 13). While these endowments offer opportunities for economic growth, infrastructural development and social progress, they also pose environmental, social and health risks when managed unsustainably. For example, emissions from South Africa's coal-fired power stations are associated with increased respiratory and cardiovascular disease and premature death (14). Studies across sub-Saharan Africa have indicated that fossil-fuel-based growth intensifies environmental degradation and air pollution, with knock-on effects on mortality and morbidity (15, 16). On the other hand, increased renewable energy consumption is associated with better health outcomes, including longer life expectancy and lower maternal and under-five mortality (17).

The nexus between either clean energy, natural resources, economic development and sustainable healthy living is neither automatic nor linear, it is deeply conditioned by the quality of a country's institutions (18, 19). Institutional quality plays a critical role in influencing regulatory enforcement, redistributive capacity, economic empowerment and the management of environmental and health externalities (20). It mediates how resource rents are managed, how environmental and health regulations are enforced, and how equitably the benefits of clean energy transitions are distributed. These institutional characteristics coexist in South Africa with significant health inequalities, reflected in high disease burdens and disparities in quality of life (54). Thus, it is theoretically and empirically plausible to integrate the roles of energy systems, resource dependence and institutional quality in analyzing sustainable healthy living outcomes in South Africa.

This study therefore examines the effects of access to clean energy, natural resource endowment, and institutional quality on sustainable healthy living outcomes in South Africa. Specifically, it (i) explores the direct effect of access to clean energy, natural resources endowment and institutional quality on DALYs (all causes) and quality of life across various levels of the health outcomes, (ii) examines how institutional quality may moderate the effects of access to clean energy and natural resources endowment on sustainable healthy living, and (iii) examines the direction and strength of the predictive relationships across the conditional distribution.

The study makes the following contributions: First, it integrates disability-adjusted life years and quality of life as comprehensive measures of sustainable healthy living, rather than focusing on aggregated health indicators such as average life expectancy, under-five mortality or broad health expenditure (62) that overlook disparities across population groups and may underestimate health inequalities and policy-relevant vulnerabilities. Second, this study advances the literature by introducing institutional quality as a moderating factor in the energy–health and natural resources–health nexus. By incorporating the moderating terms, the study demonstrates that institutional quality is not just an exogenous variable but a structural factor that conditions the effects of energy access and natural resource endowments on sustainable health outcomes. Moreover, while institutions are empirically acknowledged for their influence on environmental and economic performance (21), their role in amplifying or weakening the health benefits of clean energy and natural resources remains underexplored in empirical health studies. This study fills this gap by providing empirical evidence on these moderated pathways.

Third, the study makes a methodological contribution by employing quantile regression to uncover heterogeneous and asymmetric effects of clean energy access, natural resource endowment, and institutional quality across the entire distribution of health outcomes (low, median and upper levels) of DALYs and QTYL. This is a departure from prior studies in South Africa that rely on mean-based techniques, descriptive statistics and reviews (17, 22–25, 62) which obscure important distributional dynamics. Additionally, the study reveals quantile-specific causal linkages among the variables by applying the non-parametric Granger causality in quantiles (NpGCQ), a technique rarely used in health-energy research in Africa, The NpGCQ approach is innovative in the health-energy research in Africa as it identifies asymmetric and distribution-sensitive predictive relationships that are masked by conventional mean-based causality tests.

The rest of the study is organized as follows; section 2 presents the literature on the relationship between the determinants and the target variable. Section 3 captures the materials and methods, section 4 focuses on the results and section 5 concludes the study.

## 2 Literature review

This section focuses on the theoretical underpinnings of the study and provides a review of the empirical studies relating to the effect of effects of energy systems, natural resources and institutional quality on health outcomes.

### 2.1 Theoretical underpinnings

This study is anchored on an integrated theoretical framework combining the Grossman Health Production (GHP) model, New Institutional Economics (NIE), and the Resource Curse Hypothesis (RCH) to explain the relationships between clean energy, natural resources, institutional quality, and health outcomes.

The Grossman (8) model conceptualizes health as a durable capital stock that individuals and societies invest in through healthcare, environmental quality, and socio-economic conditions. In this context, access to clean energy improves health by reducing exposure to air pollution and also burn injuries, enhancing productivity, and improving living standards (26). The New Institutional Economics (20, 27) extends this framework by emphasizing that institutions shape economic outcomes and policy implementation. Quality institutions improve governance, reduce corruption, and enhance the delivery of public goods such as energy and healthcare. Consequently, institutional quality acts as a transmission mechanism through which clean energy access and natural resource utilization affect health outcomes. Furthermore, the Resource Curse Hypothesis (28, 29) provides a theoretical explanation for the role of natural resources. While resource abundance can generate economic wealth, it can also distort the economy to such a degree that the benefit actually becomes a curse. It could foster environmental degradation and economic inefficiency due to weak governance and extractive economic models (19), which may have attendant effect on health outcomes. This suggests the importance of institutional quality in transforming resource wealth into sustainable health gains.

By leveraging these theories, the study provides a comprehensive theoretical basis for understanding how energy access, institutional quality, and natural resources jointly influence sustainable healthy living outcomes in South Africa.

### 2.2 Effects of energy systems, natural resources and institutional quality on health outcomes

An increasing corpus of recent research has associated health burdens to energy systems and its attendant consequences including air pollution and other environmental exposures (22, 23. 30–33). Raifu and Ditep (30) examined the effect of access to clean fuel and technology on health outcomes in Africa and Asia. The findings showed that access to ‘clean' fuel and technology contributes positively to improved health outcomes in both regions, highlighting the vital role of access to clean fuel and technology in improving health outcomes. Similarly, Lyu et al. (31) demonstrated that expanding access to clean energy is critical to reducing health inequities and improving sustainable outcomes in sub-Saharan Africa. Another study by COBENEFITS (32) quantified the health benefits of increasing the use of clean and renewable energy in South Africa. These benefits include an expected 25% reduction in health costs associated with the power sector by 2050 and a considerable reduction in negative health impacts. Byaro and Rwezaula (17) employed the kernel regularized least squares machine learning approach to demonstrate that an increase in access to clean and renewable energy correlates positively with health outcomes in sub-Saharan Africa.

Studies on the effects of natural resources on health outcomes provide mixed findings. For example, Katircioglu (33) examined the impact of natural resource abundance on healthcare using the income from coal, minerals, natural gas and oil as proxies. The results show that various forms of natural resource abundance exert a mix of positive and adverse, but statistically significant, effects on the healthcare sector. The results also reveal that significant changes in the healthcare sector are preceded by changes in and shocks to natural resources, thereby suggesting the need for open governance and careful resource management. Oduyemi et al. (34) analyzed the hypothesis that mineral resource abundance causes a resource curse for African oil-rich countries using the panel threshold model. The findings revealed that resource rents adversely affect health outcomes at a lower level of economic growth, while an improvement in health performance is observed at a higher growth level. Similar findings by Sarfo and Tweneboah (35) illustrated the natural resource curse hypothesis in Africa with particular emphasis on the environmental health risks. Gani (36) showed that wealth from total natural resources is not associated with reductions in under-five mortality. In addition, Langerman and Pauw (14) confirmed that emissions from coal-fired power stations increase the incidence of respiratory, cardiovascular and cardiopulmonary diseases and contribute to premature deaths in South Africa. Studies on more localized access to natural resources have also demonstrated the effects of its use, especially of energy resources, on health outcomes. Wernecke et al. (22) in a recent study on two low-income communities in Mpumalanga, found that the utilization of locally abundant solid fuels such as coal and wood significantly elevates household air pollution and respiratory health risks. Similarly, Shezi and Wright (37) examined data on home air pollution and respiratory outcomes and disclosed that domestic use and dependence on solid and polluting fuels in informal and low-income settlements is consistently linked to poor health outcomes ranging from acute respiratory infections, and other detrimental effects. Confirming this finding, Mahlangeni et al. (15) in a study on health risks of exposure to air pollution disclosed that coal-fired power plants are major sources of air pollution which impact human health.

Regarding the role of institutional quality on health outcomes, Hadipour et al. (38) in a study on the role of institutional quality measured as a composite index of control of corruption, voice and accountability, political stability, rule of law, regulatory quality, and government effectiveness on health outcomes involving 158 countries. The results revealed a negative impact of poor quality institutions on infant mortality rates and a positive impact of accessible and effective institutions on life expectancy. Ouedraogo et al. (39) showed that most dimensions of institutional quality such as control of corruption, government effectiveness, rule of law and political stability improve health outcomes in sub-Saharan Africa. Further, Kouadio (40) studied the effects of governance quality on health outcomes in West African countries, and the results showed that institutional quality significantly improved health outcomes. Moreover, Githaiga and Kilong'I (41) demonstrated a positive association between institutional quality and human capital development in sub-Saharan Africa. Saboori et al. (42) furthermore confirmed that institutional quality reduces the negative environmental effects in the Middle East and North Africa (MENA) region. Yaman and Cetin (43) reexamined the relationship between institutional quality and pollution in emerging economies, presenting evidence that more robust institutions typically mitigate environmental degradation. Further, Li et al. (44) show that environmental non-governmental organizations (ENGOs) can influence economic restructuring and environmental performance (crowding out polluting industries) through regulatory pressure and advocacy mechanisms. This broader governance perspective implies that sustainable health outcomes are shaped not only by formal institutional quality but also by the interaction between state and non-state environmental governance actors.

### 2.3 Research gap and contribution statement

In summary, the existing literature reveals that clean energy enhances sustainable healthy living (17, 30, 31), while natural resources have a negative effect on health outcomes (14, 33, 36). Institutional quality plays a critical role, influencing health outcomes and environmental quality (38–40, 42).

However, three important gaps remain. First, most existing studies (31, 33, 36, 39) focus on isolated relationships (energy–health, resources–environment, or governance–health) rather than providing an integrated framework that jointly examines these interactions. Second, limited attention has been given to comprehensive health indicators such as DALYs and the multidimensional quality of life, particularly in country-specific contexts like South Africa. Third, recent literature such as Lyu et al. (31) support the use of distribution-sensitive approaches in energy–health related studies, yet empirical evidence on asymmetric and heterogeneous effects across different health conditions remains scarce. Similarly, the moderating role of institutional quality in shaping the effectiveness of clean energy and natural resource utilization remains underexplored.

This study contributes to the literature by addressing these gaps through (i) an integrated energy–resource–institutional-health framework, (ii) the use of DALYs and quality of life as comprehensive health metrics, and (iii) the application of quantile-based and non-parametric causality approaches to capture distributional heterogeneity. The study examines both the direct and indirect effects of access to clean energy and natural resources on sustainable healthy living (DALYs and quality of life outcomes) while accounting for the moderating role of institutional quality. Further, this research applies a quantile-based regression to uncover heterogeneous and asymmetric effects across different levels of the health burden and wellbeing, thereby identifying where impacts are strongest especially amongst vulnerable or high-risk populations to help inform specific policy decisions.

## 3 Materials and methods

### 3.1 Data

This study uses quarterly data for South Africa covering the period from 2000 Q1 to 2022 Q4. The choice of the selected period is based on data availability and within the period approved by the University of South Africa's College of Human Sciences Research Ethics Committee (Reference number: 9267). The study adopts a national-level time-series design rather than a cross-sectional or provincial sampling approach. Therefore, no sub-national sampling of South African provinces or regions is involved. Instead, the analysis relies on nationally aggregated and internationally harmonized datasets that are representative of the entire South African population, consistent with prior macro-level studies on energy–health dynamics (31). The data are obtained directly from the indicated sources except institutional quality which is a construct or index of six institutional indicators of the World Governance Indicators (WGI). The annual data series was transformed and converted into a quarterly frequency using a quadratic-match-sum interpolation procedure, which preserves the annual totals or index paths while generating smooth within-year quarterly values and for avoiding the small-sample problem (57). The description of the variables are from their sources and is as follows:

Disability-adjusted life years (DALYs): this is a measure of the total healthy years lost as a result of premature death and disability from all causes. This composite indicator captures both fatal and non-fatal health outcomes, thus providing a comprehensive measure of population health burden. Higher DALY values indicate poorer health outcomes. Data are sourced from the Institute for Health Metrics and Evaluation (IHME) (58). It serves as a dependent variable.

Quality of life (QTYL): this is an index of two sustainable indicators (i) access to drinking water and sewage treatment and (ii) exposure to environmental risks such as fine particle matter with micrometer of 2.5 or less (PM2.5) (45). The index measures social welfare and human wellbeing outcomes, including health status, access to clean water and sanitation, and environmental health as the determinants of population health. It is computed by Sarkodie et al., (46) and used for testing robustness of the estimates.

Access to clean energy (ACE): this is a measure of the number of people that have access to clean cooking fuels. Clean cooking fuels and technologies represent non-solid fuels such as natural gas, ethanol or electric technologies with almost zero carbon emission. The data is from the World Bank, processed by Our World in Data (2023). It is an independent variable.

Natural assets endowment (NAE): this is a composite index comprising land, forests, water, biodiversity and climate stability, as computed by Sarkodie et al. (46). It measures the stock and quality of environmental resources available to support economic activity and human welfare. A higher NAE score indicates sustainable resource management, resulting in cleaner air, safer water, and improved food and environmental quality, which influences health and wellbeing. It is used as an independent variable to capture the ecological determinants of health outcomes.

Institutional quality (INQ): this is a composite index that captures the extent to which governance structures influence economic and social outcomes, including population health. Strong institutions promote efficient allocation of resources, social trust, and policy implementation, all of which are essential for a functional health system. In this study, institutional quality is measured using Principal Component Analysis (PCA), a data reduction technique widely applied in empirical governance studies to construct composite indices. The PCA approach reduces dimensionality by extracting the common variation from multiple correlated governance indicators. Specifically, six dimensions from the World Governance Indicators (WGI) database are employed: control of corruption (CC), government effectiveness (GE), political stability and absence of violence/terrorism (PV), rule of law (RL), regulatory quality (RQ), and voice and accountability (VA) (56). The PCA dimensions for INQ are expressed in Equation 1 as:


$$\begin{array}{l} INQ=\theta_{1}CC+\theta_{2}GE+\theta_{3}PV+\theta_{4}RL+\theta_{5}RQ+\theta_{6}VA \end{array}$$ (1)

**Figure 1:**
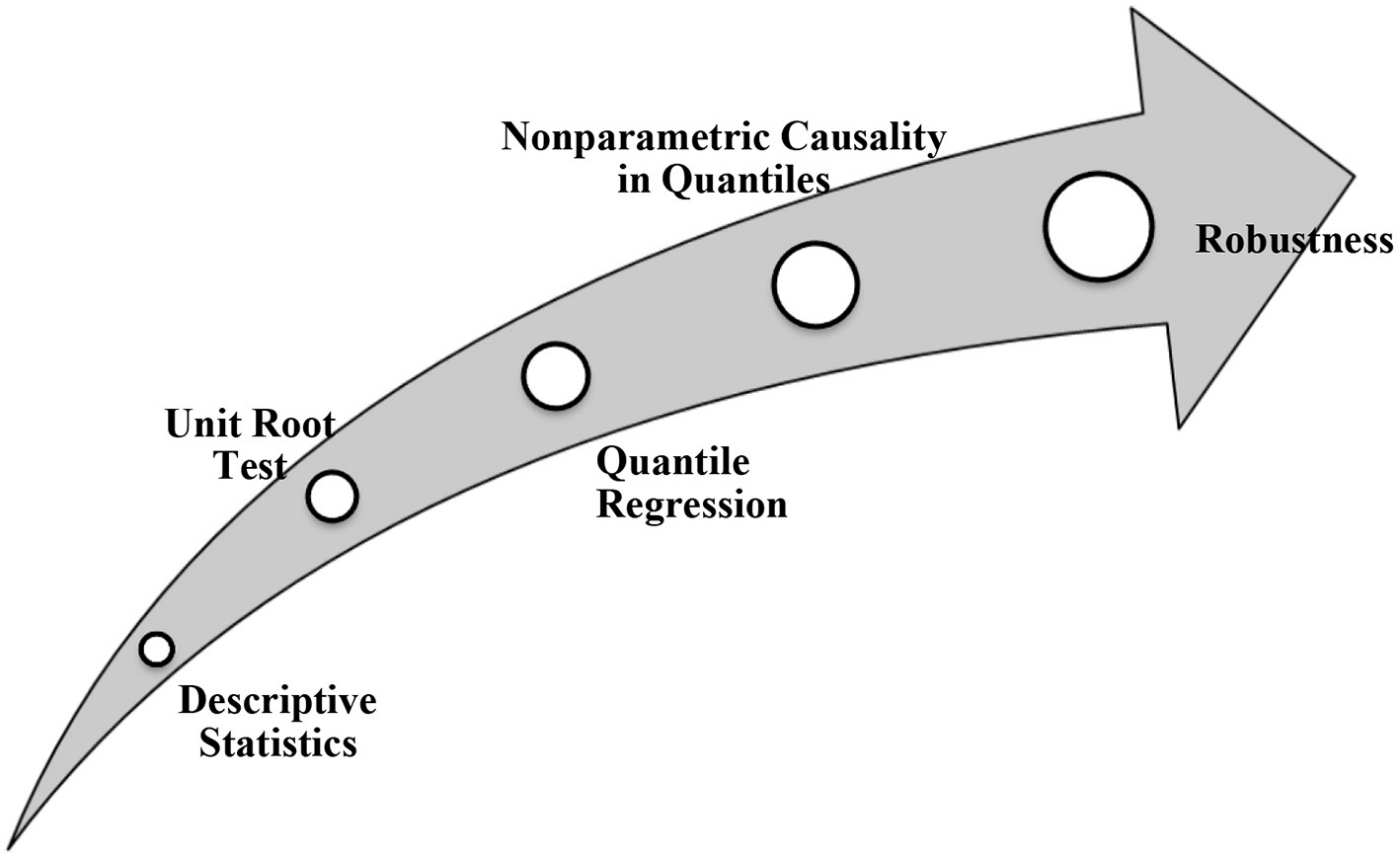
Estimation algorithm.

Where θ represent the weights. The detailed PCA results, including eigenvalues, factor loadings, and sampling adequacy statistics, are presented in Appendix Table 1A and Figure 1A for full transparency and replicability.

### 3.2 Model specification and methods

In this section, we specify the model and present the methods used for the data analysis. The stochastic form of the relationship between the sustainable health outcomes and the factor variables (access to clean energy (ACE), natural resources (NAE) and institutional quality (INQ)) is given by Equation 2 as follows:


$$\begin{array}{l} Health\;Outcome=\;\varphi +\alpha_{1}ACE_{t}+\alpha_{2}NAE_{t}+\alpha_{3}INQ_{t}+\varepsilon_{t} \end{array}$$ (2)

Where *Health Outcome* is proxied by *DALYs* and *QTYL* for Disability-Adjusted Life Years from communicable, maternal, neonatal, injury and nutritional diseases, and quality of life respectively.

For the moderating effect, we incorporate the interactive terms (ACE^*^INQ_*t*_ and NAE^*^INQ_*t*_) to capture how institutional quality influences the effect of ACE and NAE on sustainable health outcomes. The relationship is expressed as follows:


$$\begin{array}{l} Health\;Outcome=\;\delta +\beta_{1}ACE_{t}+\beta_{2}ACE^{*}INQ_{t}+\beta_{3}NAE_{t}\\ \;+\beta_{4}NAE^{*}INQ_{t}+\;\beta_{5}INQ_{t}+\epsilon_{t} \end{array}$$ (3)

Equation 3 describes the quantile regression method used in estimating the relationships. Then, the novel Non-parametric Granger Causality in Quantiles (NpGCQ) is applied to evaluate the predictive effect of the determinants across the conditional distribution of the health outcome indicators. The null and alternative hypotheses of the test are shown in Equations 4 and 5 respectively. Figure 1 captures the estimation algorithm.

### 3.3 Quantile regression

The Quantile Regression (QR) technique introduced by Koenker and Bassett (59) examines the impact of an independent or factor variable *X* on the γ-th conditional quantile of a dependent variable *Y*. The standard QR between an independent variable *X* and a dependent variable *Y* is expressed as:


$$\begin{array}{l} QR\left(Y_{\gamma }|X\right)=\alpha \left(Y_{\gamma }\right)+\theta \left(Y_{\gamma }\right)X \end{array}$$ (4)

Where α and θ stand for the estimated intercept term and the slope coefficients between the explanatory variable *X* and the conditional quantiles γ of the dependent variable *Y*.

Recent methodological advances have extended quantile regression frameworks to more complex panel structures, including models with latent group heterogeneity and functional coefficients (47). While such approaches offer flexibility in capturing cross-sectional heterogeneity, the present study adopts a standard quantile regression consistent with Koenker and Bassett (59) which is robust to distributional assumptions. This ensures interpretability within the context of South Africa's aggregated data structure.

### 3.4 Non-parametric granger causality in quantile

The Non-parametric Granger Causality in Quantiles (NpGCQ) approach developed by Balcilar et al. (60) and recently applied by Iorember et al. (48) to evaluates the predictive relationships in quantiles between the means and variances of an independent or factor variable *x* and a dependent variable *y* at the ϑ-th conditional quantile. The null hypothesis (*H*_*Null*_) and alternative hypothesis (*H*_*ALT*_) of the NpGCQ approach is given by Equations 5 and 6 respectively.


$$\begin{array}{l} H_{Null}:\;P\left\{F_{y_{t}*|Z_{t-1}}\left\{\vartheta (y_{t-1})|Z_{t-1}\right\}=\vartheta \right\}=1,\;*=1\;and\;2 \end{array}$$ (5)

$$H_{ALT}:\;P\left\{F_{y_{t}*|Z_{t-1}}\left\{\vartheta (y_{t-1})|Z_{t-1}\right\}=\vartheta \right\}\text{ < }1,\;*=1\;and\;2$$ (6)

where *Z* represents a vector of *y* and *x*, ϑ stands for the conditional quantiles, and ^*^ captures the moments. The non-parametric NpGCQ of the factor variable *x* and dependent variable *y* at the ϑ-th quantile is tested with the moment ^*^ value 1 and 2, respectively. Figure 1 displays the estimation algorithm.

## 4 Results

### 4.1 Descriptive statistics

The descriptive statistics in Table 1 show a summary and characteristics of the variables based on 92 observations. All the variables have mean and median values that are close to each other. This means that there is not much central-tendency distortion. DALYs has a low standard deviation (0.0833), which means it is stable. QTYL has a higher but less than 1 variability (0.8764), indicating that the quality-of-life scores are more likely to change. ACE and NAE also have moderate variability (0.2292 and 0.2899) respectively. INQ has a higher standard deviation (0.7933), suggesting noticeable variation in institutional quality. The skewness values are mostly low, indicating mild asymmetry. For example, DALYs and NAE are slightly positively skewed, while QTYL and ACE are slightly negatively skewed. Kurtosis values close to 3 mean that the distributions are close to normal. The only exception is NAE, which has a higher kurtosis value (3.9354), indicating a more peaked distribution. In general, the results show that the variables vary moderately with mild skewness and mixed evidence of normality. This validates the application of quantile regression, as it yields resilient estimates that are independent of normality assumptions and can identify heterogeneous effects across the distribution of health outcomes.

**Table 1:** Descriptive and summary statistics.

Statistics	DALYs	QTYL	ACE	NAE	INQ
Mean	10.5498	−1.7053	17.5137	−0.5119	−0.2599
Median	10.5323	−1.4932	17.5462	0.5075	−0.4438
Maximum	10.7145	−0.7566	17.8449	0.3212	1.0675
Minimum	10.4143	−3.2969	17.0728	−1.1622–	−1.6659
Std. Dev.	0.0833	0.8764	0.2292	0.2899	0.7933
Skewness	0.4249	−0.4084	−0.3315	0.3597	0.2830
Kurtosis	1.9603	1.5724	1.8759	3.9354	2.0135
Jarque-Bera	6.9118	10.370	6.5287	5.3377	4.9581
Probability	0.0316	0.0056	0.0382	0.0693	0.0838
Observations	92	92	92	92	92

DALYs, disability-adjusted life years; QTYL, quality of life; ACE, access to clean energy; NAE, natural assets endowment; INQ, institutional quality.

### 4.2 Unit root test

The quantile unit root results in Table 2 provide evidence of stationarity across the conditional distribution depending on whether the t-statistic is more negative than the corresponding critical value, a necessary condition for rejecting the null hypothesis of no stationarity. For the health outcome variables (DALYs and QTYL), stationarity is mainly observed in the middle and higher quantiles respectively, where the t-statistics are more negative than the critical values (CVs). This suggests that these health outcomes primarily revert to mean under moderate to severe health burdens, respectively. ACE exhibits strong and consistent stationarity across nearly all quantiles as the t-statistics are substantially more negative than the corresponding critical values across the quantiles, indicating stable long-run behavior. NAE also shows stationarity in several quantiles, particularly in the middle, while INQ shows a unique pattern, with stationarity emerging more clearly in the higher quantiles. Overall, the results reveal that stationarity is quantile-dependent, which reinforces the use of quantile-based approaches, such as quantile regression and non-parametric quantile Granger causality, to capture heterogeneous dynamics across the distribution.

**Table 2:** Quantile unit root test.

Quantile	DALYs		QTYL		ACE		NAE		INQ	
	t-stat	CV	t-stat	CV	t-stat	CV	t-stat	CV	t-stat	CV
0.050	1.675	−14.313	0.557	−14.841	−5.505	−11.492	0.439	−6.647	−0.927	−12.803
0.100	0.630	−7.012	0.515	−6.777	−9.054	−4.740	−1.114	−4.381	−0.539	−6.659
0.150	−0.376	−4.076	1.643	−4.350	−7.634	−3.208	0.096	−4.047	0.099	−4.460
0.200	−0.792	−2.995	2.787	−3.051	−7.239	−2.509	0.331	−4.427	0.385	−3.388
0.250	−0.781	−1.792	1.980	−1.878	−8.628	−1.106	−1.774	−4.377	1.239	−2.376
0.300	−1.659	−0.700	1.658	−1.148	−12.894	−0.350	−3.757	−3.626	1.611	−1.585
0.350	−2.527	−0.703	2.350	−1.180	−14.226	−0.620	−3.605	−2.765	1.421	−1.266
0.400	−2.970	−1.778	0.143	−1.269	−17.032	−4.403	−2.297	−2.756	1.871	−1.537
0.450	−3.658	−3.519	0.122	−1.297	−17.572	−13.957	−3.134	−1.606	0.679	−1.673
0.500	−3.846	−3.128	−0.167	−1.479	−20.801	−18.389	−1.249	−4.387	0.196	−1.786
0.550	−5.328	−2.604	−0.530	−1.761	−21.598	−18.894	0.979	−4.398	−0.303	−1.784
0.600	−4.386	−2.384	−1.998	−2.049	−25.046	−12.918	0.877	−4.350	−0.624	−1.526
0.650	−2.734	−2.169	−1.365	−2.314	−28.630	−0.740	0.639	−4.243	−1.159	−1.314
0.700	−2.321	−1.806	−1.094	−2.463	−29.251	−0.231	0.089	−4.840	−1.360	−1.399
0.750	−1.663	−1.744	−1.360	−3.172	−34.417	−0.831	−0.757	−4.876	−2.127	−1.815
0.800	−1.216	−2.372	−1.059	−4.694	−14.391	−2.737	−3.268	−5.652	−2.644	−2.598
0.850	−1.808	−3.121	−4.609	−4.492	−19.625	−3.600	−1.567	−5.611	−3.864	−3.705
0.900	−2.364	−4.722	−2.365	−1.382	−13.090	−6.475	−1.207	−5.903	−1.148	−6.101
0.950	−1.950	−12.265	−5.714	−4.961	−8.894	−17.099	−0.976	−8.522	0.692	−33.074

DALYs, disability-adjusted life years; QTYL, quality of life; ACE, access to clean energy; NAE, natural assets endowment; INQ, institutional quality.

### 4.3 Main analysis

This section presents the main results of the study. Table 3 displays the asymmetric effects of ACE, NAE and INQ on both proxies of sustainable health outcomes. The top part of Table 3 shows the direct effect and the bottom part reveals the moderating effect.

**Table 3:** Results of the quantiles regression showing the asymmetric effect.

Variable	Disability-adjusted life years (DALYs)			Quality of life (QTYL)		
	Lower 0.25	Middle 0.50	Upper 0.75	Lower 0.25	Middle 0.5	Upper 0.75
Direct effect						
ACE	−0.4174^***^	−0.3857^***^	−0.3525^***^	6.4788^***^	3.3711^**^	3.2953^***^
NAE	−0.0024	0.0017	0.0249	0.2959	−0.1464	−0.3702
15.5-7.4,-15.5175.3mmINQ	−0.0064^**^	−0.0014	0.0030	0.7924^**^	0.0961	0.1917
Moderating effect						
ACE	−4.225^***^	−0.4227^***^	−0.4079^***^	6.1243^***^	4.9366^***^	2.7276^***^
NAE	−0.0007	−0.0067	0.0054	−0.5195	−1.4880^***^	−1.1262^***^
INQ	0.2002	0.6302^*^	1.5239^***^	−2.5444^***^	1.5918	−1.2359
**ACE** ^*^ **INQ**	**0.0119**	**−0.0371** ^**^	**−0.0893** ^***^	**−1.4163**	**−0.9005**	**0.6871**
**NAE** ^*^ **INQ**	**0.0025**	**−0.0198** ^*^	**−0.0412** ^***^	**−0.1919**	**−0.8428** ^**^	**−0.7894** ^***^

( )denotes standard errors.

^***^p < 0.01, ^**^p < 0.05, ^*^p < 0.1.

That is, ^***^, ^**^, ^*^ imply statistical significance at 1, 5 and 10% respectively.

DALYs, disability-adjusted life years; QTYL, quality of life; ACE, access to clean energy; NAE, natural assets endowment; INQ, institutional quality; ACE^*^INQ, moderating effect of INQ on ACE; NAE^*^INQ, moderating effect of NAE on ACE. The bold value indicates moderating effect.

### 4.4 Direct effect

The quantile regression results in Table 3 presents the main results of the study. It displays the asymmetric effects of ACE, NAE and INQ on the two measures of sustainable health outcomes–DALYs and QTYL at the lower (0.25), middle (0.50), and upper (0.75) quantiles. The top part of Table 3 shows the direct effect and the bottom part reveals the moderating effect of institutional quality. For the DALYs model, the results show that ACE has a strong and statistically significant negative effect on DALYs in all quantiles, with coefficients of −0.4174, −0.3857, and −0.3525, respectively. NAE, on the other hand, shows no statistically significant effect in any quantiles. Although it has a marginal but non-significant effect at the lower quantile. The results for INQ indicate a marginal and statistically significant negative effect (−0.0064) at the lower quantiles, implying that enhanced institutional quality alleviates the disease burden predominantly among populations with relatively low DALYs. The effects at the median and higher quantiles are not significant. In all, the results of the DALYs model show that access to clean energy is the most reliable and influential factor in reducing disability-adjusted life years and improving health outcomes.

Regarding quality of life (QTYL), the quantile regression also exhibit substantial asymmetry across the quantiles distribution. ACE has a strong, positive (increasing) and statistically significant effect on QTYL at all three quantiles, with coefficients of 6.4788 at lower, 3.3711 at median, and 3.2953 upper quantiles respectively. NAE exhibits no statistically significant effect on QTYL across all quantiles. INQ however, shows a positive and statistically significant effect only at the lower quantile (0.7924), indicating that improved governance and institutional performance meaningfully enhance the quality of life among the most vulnerable or low–wellbeing groups. The effects at the middle and upper quantiles are positive but statistically insignificant.

### 4.5 Moderating effect of institutional quality

The results of the moderating effect show how INQ influences the effects of ACE and NAE on the health outcome variables across the quantiles distribution. The interactions terms ACE^*^INQ and NAE^*^INQ show the moderating effects of INQ on ACE and NAE respectively. For the DALYs model, ACE^*^INQ capturing the moderating effect of INQ on the relationship between ACE and DALYs is negative (reducing) and statistically significant at the middle (−0.0371) and upper (−0.0893) quantiles but a small and insignificant effect at the lower quantile. Similarly, the results of the interactive term (NAE^*^INQ) show significant reducing effect on DALYs from the middle (−0.0198) to upper (−0.0412) quantiles, with no significant effect at the lower quantiles.

For the QTYL model, the interactive term ACE^*^INQ reveals a non-statistical significance across the quantiles. It shows negative and significant coefficients at the lower and middle quantiles and a positive but non-significant effect at the upper quantile. This pattern indicates that institutional quality diminishes the consistently positive and highly significant direct effect of ACE on QTYL across all quantiles. More so, the interaction term NAE^*^INQ is negative for all quantiles, and it is statistically significant at the middle (−0.8428) and upper (−0.7894) quantiles. This indicates that institutional quality exacerbates the detrimental impact of natural resource endowment on QTYL. That is, rather than balancing out the reducing effects of NAE on QTYL, the institution quality makes the negative effects of depending on natural resources for quality of life even worse.

### 4.5 Robustness checks using the non-parametric granger causality in quantiles

**Figure 2:**
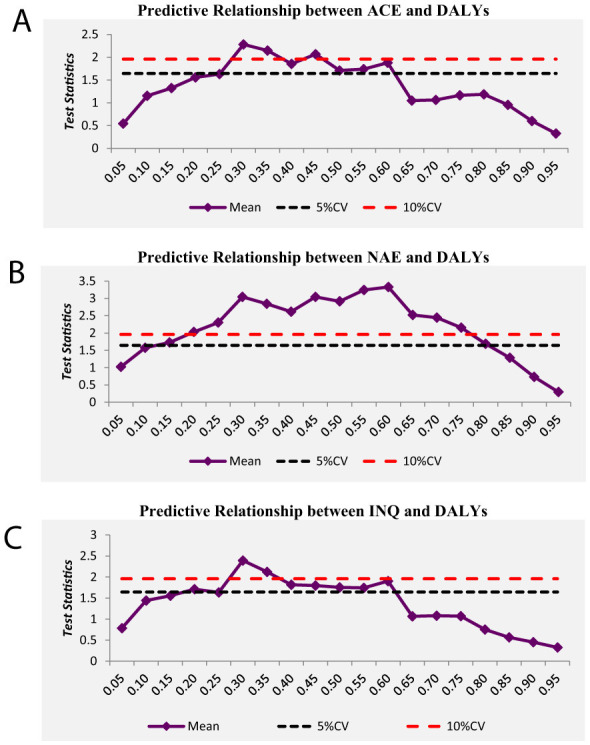
**(A–C)** Non-parametric granger causality in quantiles (DALYs).

**Figure 3:**
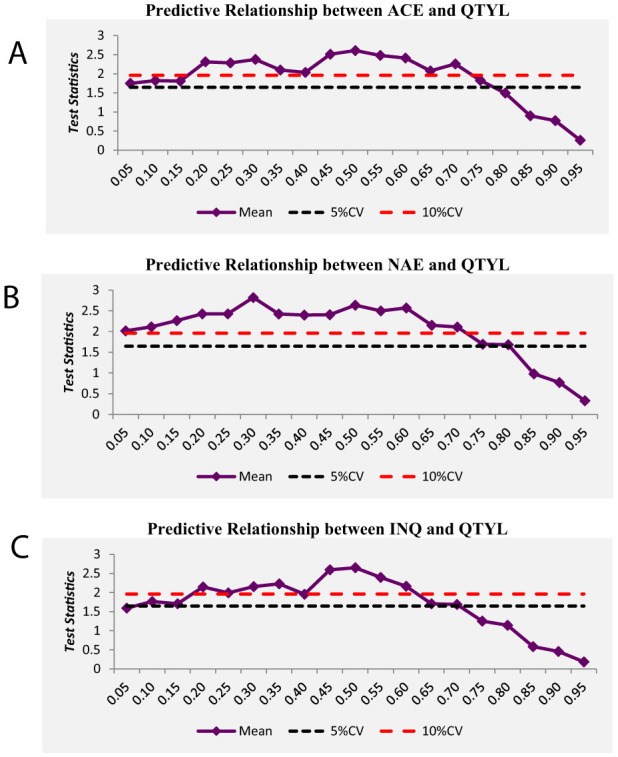
**(A–C)** Non-parametric granger causality in quantiles (QTYL).

To validate the quantile regression findings, we apply the Non-parametric Granger Causality in Quantiles (NpGC) for both the DALYs and QTYL models and the results are shown in Figures 2, 3 respectively. For both figures, the purple line represents the test statistic of the NpGC test at each quantile (γ = 0.050.95). The black and red dashed lines represent 5% and 10% significant values thresholds respectively. If the purple line is above these thresholds, the null hypothesis of no predictive effect is rejected, implying that the existence of predictive effect or relationship at that quantile.

For the DALYs model in Figure 2A, the purple line is above the 5% significance threshold across 0.25 to 0.60 quantiles, suggesting that the null hypothesis of no predictive association or relationship between ACE and DALYs is rejected across those quantiles. The highest test statistics are around the lower-middle quantiles, indicating that ACE Granger causes DALYs mostly at the lower and middle quantiles representing population group with moderately low health risk conditions. These findings are consistent with the earlier findings of the quantile regression. Although at the extreme quantiles representing healthiest and most health-stressed population groups, the results indicate no predictive relationship. Similarly the results in Figure 2B provides evidence to reject the null hypothesis of no predictive relationship between NAE and DALYs. It displays a significant humped shaped association between NAE and DALYs from around 0.15–0.80 quantiles. More so, Figure 2C reveal that, the null hypothesis of no predictive effect from INQ to DALYs is rejected across (0.25–0.60) quantiles with highest statistics at the lower quantiles. These findings align with the earlier findings, thereby confirming the robustness of the estimates.

For the QTYL model in Figure 3A, the results show that the null hypothesis of no predictive relationship between ACE and QTYL is rejected around 0.15–0.75 quantiles. The effect is highly uneven across the quantiles suggestive of asymmetric patterns. Similarly, the null hypothesis of no predictive relationship between NAE and QTYL is rejected around 0.05–0.80 quantiles in Figure 3B with clear evidence of asymmetry effect across the quantiles. More so, the null hypothesis is rejected for the predictive relationship between INQ and QTYL around 0.10–0.70 quantiles in Figure 3C. The relationship is highly asymmetric with the effect peaking at the middle quantiles.

## 5 Discussion

This study set out to examine the asymmetric effects of access to clean energy and natural resource endowments on sustainable healthy living measured by DALYs and quality of life in South Africa, while accounting for the direct and indirect roles of institutional quality. The results provide compelling evidence that increasing access to clean energy is the most consistent and important of these factors in improving health outcomes.

Access to clean energy is significantly associated with reductions in DALYs and improvements in quality of life across all quantiles, indicating that energy access policies if effectively implemented will have broad-based health benefits across both vulnerable and relatively healthier population groups. This is consistent with global and regional positions on the health and productivity benefits of using clean or renewable energy. The current findings imply that expanding access to clean cooking fuels and electricity should be prioritized as a public health intervention rather than solely an energy policy objective, as in the case made in South Africa for the removal of fossil fuels for use in domestic settings (26). Specifically, targeted subsidies for clean cooking technologies (e.g., LPG, electricity, and solar solutions), investments in decentralized renewable energy systems (such as mini-grids in underserved communities), and scaling up rural and informal settlement electrification programs can significantly reduce the disease burden and improve quality of life, especially in energy impoverished communities. In addition, the effectiveness of these interventions can be enhanced through the development of green finance mechanisms and urban green technology transfer networks, which facilitate the diffusion of clean technologies and mobilize capital for sustainable energy investments (49). Such mechanisms not only accelerate clean energy adoption but also indirectly contribute to improved health outcomes by reducing environmental risks and enhancing energy accessibility.

The findings corroborate previous studies (17, 30, 31) which established a correlation between clean or renewable energy and health outcome measures such as life expectancy, maternal and under-five mortality, disability, respiratory illness, and out-of-pocket health expenditure. The stronger effects observed at lower health quantiles of DALYs and quality of life suggest that energy access policies targeted at both the health advantaged and vulnerable households have substantial benefits for health equity and inclusive development in South Africa. The finding aligns with those of Koomson (23) and Shupler et al. (24), who observed the detrimental impact of energy deprivation on the physical and mental wellbeing of poor and vulnerable households. The finding is particularly significant for the sustainable development goals, as it emphasizes the crucial role of energy access (SDG 7) in improving health outcomes (SDG 3) and reducing poverty-related vulnerabilities (SDG 1).

Conversely, the lack of a direct significant effect of natural resource endowment on both DALYs and quality of life across all quantiles of the distribution, implies that natural resource endowment does not directly translate into better living standards or wellbeing. The findings suggest the challenge of turning South Africa's wealth of resources into real welfare improvements, consistent with deep-seated challenges of service delivery, mis-governance, corruption and state capture as reported by the Zondo Commission of inquiry (50). In line with Oduyemi et al. (34), and Sarfo and Tweneboah (35), the findings indicate that resource exploitation may persist in generating environmental and health liabilities without enhancing community wellbeing, exemplifying a resource-curse hypothesis in South Africa. This also corroborates the findings of Kansheba and Marobhe (51), which observed that resource endowments in sub-Saharan Africa have generally turned out to be a curse as a result of poor institutions characterized by widespread corruption and undemocratic government processes.

Further, the current findings show that institutional quality exhibits asymmetric and heterogeneous effects, reducing DALYs and improving quality of life at the lower levels of the health distribution. This aligns with the findings of Ouedraogo et al. (39) and Hadipour et al. (38), who showed that well-functioning institutions enhance health outcomes. Overall, the results of this direct effect show substantial asymmetry across the distributions. It shows that institutional quality matters primarily for the most vulnerable population subgroups, while natural resource endowment reveals no significant influence on sustainable health outcomes, suggesting the need for enhanced strategies and mechanisms to translate the nation's wealth toward improving sustainable healthy living.

The moderating effect of institutional quality deepens these findings. The interaction between institutional quality and access to clean energy indicates that governance quality enhances the effectiveness of energy policies in improving health outcomes. This suggests that energy transition strategies must be embedded and even prioritized within strong institutional frameworks to achieve maximum impact and deliver the indicated energy access benefits. More so, quality institutions help mitigate the harmful effects of depending on natural resources, especially from the middle to the upper levels of the health distribution. According to the Natural Resource Governance Framework (52), the mechanisms by which state power and government responsibilities relating to natural resources are exercised can ensure effective management of resource revenues and value creation for the betterment of the economy and social livelihoods of people. Effective governance and regulatory quality improve resource revenue management, enabling greater investment in health infrastructure, environmental protection, and social services. This resonates with the findings of Hadipour et al. (38) and Ouedraogo et al. (39) which have also shown that institutional quality is a positive correlate of health outcomes in sub-Saharan Africa. However, for the quality of life effect, the results show that institutional quality weakens the positive effects of access to clean energy on life quality across the health distribution and further deepens the deteriorating effect of natural resource endowment on quality of life. This suggests that governance frameworks may not yet be optimized to ensure that improvements in resource revenues translate into sustainable healthy gains. The findings correspond with concerns raised by Yaman and Cetin (43) about governance failures exacerbating pollution-related inequality.

## 6 Conclusion and implications

This study examined the asymmetric and moderated effects of access to clean energy, natural resource endowment, and institutional quality on sustainable healthy living outcomes measured by DALYs and quality of life in South Africa. Utilizing quantile regression and non-parametric Granger causality within quantiles, the findings indicate that access to clean energy is the most important determinant in decreasing DALYs from all causes and enhancing quality of life. The results indicate that the highest benefits of access to clean energy are realized by communities facing significant health challenges and substandard living conditions. Natural resource endowment has no direct effect on DALYs and QTYL, suggesting governance and distributional challenges in converting resource wealth into welfare advantages. Institutional quality has an asymmetric but important effect. It improves health outcomes mainly for the most vulnerable groups and boosts the good health effects of having access to clean energy. However, it exerts a limited and asymmetric ability to turn natural resource rents into increases in quality of life.

Further, the moderating term of institutional quality and access to clean energy significantly strengthens the reducing effect of access to clean energy on DALYs for moderate to highly vulnerable population groups. Additionally, institutional quality helps mitigate the harmful effects associated with resource dependence for moderate to highly vulnerable population groups. However, for the quality of life effect, the results show that institutional quality weakens the positive effects of access to clean energy on life quality across the health distribution and further deepens the deteriorating effect of natural resource endowment on quality of life.

The results point to several detailed and actionable policy implications. First, the South African government should accelerate clean energy adoption by: (i) expanding investments in renewable energy infrastructure (solar, wind, and hybrid systems); (ii) scaling up clean cooking initiatives; and (iii) providing targeted subsidies and financial support to low-income households. These measures can significantly reduce indoor and outdoor air pollution, but also other health and injury outcomes, and the associated disease burdens. Second, natural resource management policies should be redesigned to ensure that resource revenues are effectively invested in health-promoting sectors. This can be achieved through sovereign wealth funds, earmarked public spending on healthcare and clean energy, and stronger monitoring of resource utilization. Third, improvements to institutional quality may require specific governance reforms, including: (i) strengthening anti-corruption frameworks in the energy and natural resource sectors; (ii) enhancing regulatory enforcement of environmental and energy standards; and (iii) improving transparency and accountability in public resource allocation. These reforms will ensure that energy and resource policies translate into tangible health benefits.

Although the study makes important contributions relevant to South Africa's development plan, it relies on aggregated national-level data measures which may obscure important sub-national disparities. This is particularly important for South Africa where access to energy, health services, and socio-economic dynamics varies substantially across population groups, communities and provinces which may affect generalization. Therefore, future studies may consider using disaggregated data to avoid understating localized vulnerabilities or overgeneralizing national trends.

## Figures and Tables

**Figure A1:**
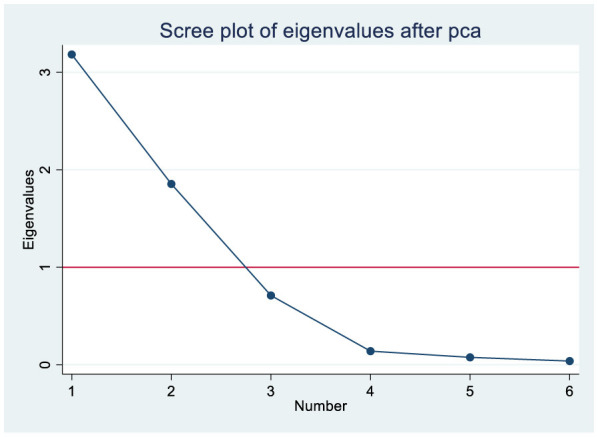
In computing the INQ index, the first step is the correlation matrix which revealed strong positive relationships among the indicators, suggesting that they measure related. The eigenvalue results show that the component 1 (Compt 1) has an eigenvalue of 3.1820, explaining 53.03% of the total variance. The remaining components jointly explain less than 50% of the variance. The Compt loadings are mostly high and positive for most variables, indicating that the institutional indicators contribute to the INQ composite index. The component loadings further provide insight into the relative importance of each governance indicator in constructing the institutional quality index. Control of corruption and government effectiveness exhibit the highest loadings, indicating that they are the dominant contributors to institutional quality. Regulatory quality and voice and accountability show moderate contributions, while rule of law also plays a meaningful but relatively smaller role. In contrast, political stability contributes minimally and negatively, suggesting that it does not significantly drive variations in institutional quality within the study period. The Kaiser–Meyer–Olkin (KMO) statistic of 0.6283, which is above the 0.50 threshold, confirms sampling adequacy and suitability for PCA (53). Therefore, the derived INQ index is a good representation of the governance variables. Figure 1A in the Appendix shows the screen plot of eigenvalues.

## Data Availability

The raw data supporting the conclusions of this article will be made available by the authors, without undue reservation.

## Appendix

**Table A1:** Results of the principal component analysis for INQ index construction.

Variable	CC	GE	PV	RL	RQ	VA
Panel (A) Correlation matrix						
CC	1.000					
GE	0.9229	1.000				
PV	–0.1031	–0.0241	1.000			
RL	0.4169	0.4140	0.0360	1.000		
RQ	0.6964	0.7511	0.4446	0.4831	1.000	
VA	0.6578	0.5606	–0.7394	0.2492	0.0366	1.000
	**Compt 1**	**Compt 2**	**Compt 3**	**Compt 4**	**Compt 5**	**Compt 6**
Panel B: Principal components						
Eigenvalue	3.1820	1.8539	0.7104	0.1396	0.0759	0.0381
Proportion	0.5303	0.3090	0.1184	0.0233	0.0127	0.0063
**Variable**	**Loading**
Panel C: Component loadings (compt 1)	
CC	0.5370
GE	0.5310
PV	-0.0635
RL	0.3375
RQ	0.4221
VA	0.3655
**Statistic**	**Value**
Panel (D) Sampling adequacy and suitability	
Kaiser–Meyer–Olkin (KMO)	0.6283
